# Changes in Neuronal Representations of Consonants in the Ascending Auditory System and Their Role in Speech Recognition

**DOI:** 10.3389/fnins.2018.00671

**Published:** 2018-10-12

**Authors:** Mark A. Steadman, Christian J. Sumner

**Affiliations:** ^1^MRC Institute of Hearing Research, School of Medicine, The University of Nottingham, Nottingham, United Kingdom; ^2^Department of Bioengineering, Imperial College London, London, United Kingdom

**Keywords:** auditory nerve, inferior colliculus, auditory cortex, speech processing, neural coding, spike timing

## Abstract

A fundamental task of the ascending auditory system is to produce representations that facilitate the recognition of complex sounds. This is particularly challenging in the context of acoustic variability, such as that between different talkers producing the same phoneme. These representations are transformed as information is propagated throughout the ascending auditory system from the inner ear to the auditory cortex (AI). Investigating these transformations and their role in speech recognition is key to understanding hearing impairment and the development of future clinical interventions. Here, we obtained neural responses to an extensive set of natural vowel-consonant-vowel phoneme sequences, each produced by multiple talkers, in three stages of the auditory processing pathway. Auditory nerve (AN) representations were simulated using a model of the peripheral auditory system and extracellular neuronal activity was recorded in the inferior colliculus (IC) and primary auditory cortex (AI) of anaesthetized guinea pigs. A classifier was developed to examine the efficacy of these representations for recognizing the speech sounds. Individual neurons convey progressively less information from AN to AI. Nonetheless, at the population level, representations are sufficiently rich to facilitate recognition of consonants with a high degree of accuracy at all stages indicating a progression from a dense, redundant representation to a sparse, distributed one. We examined the timescale of the neural code for consonant recognition and found that optimal timescales increase throughout the ascending auditory system from a few milliseconds in the periphery to several tens of milliseconds in the cortex. Despite these longer timescales, we found little evidence to suggest that representations up to the level of AI become increasingly invariant to across-talker differences. Instead, our results support the idea that the role of the subcortical auditory system is one of dimensionality expansion, which could provide a basis for flexible classification of arbitrary speech sounds.

## Introduction

As a prerequisite for speech recognition, the early auditory system must be sensitive to acoustic cues that differentiate phonemes, the fundamental units of speech. For example, the vowel sounds */a/* and /*i*/ distinguish the word “had” from “hid” and the consonants */d/* and */t/* distinguish the word “hid” from “hit.” This sensitivity begins in the cochlea, where sounds are transduced from airborne vibrations to patterns of electrical activity in the auditory nerve (AN). These patterns are subsequently transformed as they propagate throughout the ascending auditory system to form the basis of speech recognition in the brain.

The classical approach to investigate the neural basis speech recognition is to define an acoustic cue for a given set of phoneme contrasts. One example of this is the voice-onset time (VOT), which indicates the distinction between phonemes articulated in a similar way, such as */b/* vs */p/, /d/* vs */t/*, or */g/* vs */k/*. This cue appears to be reliably represented throughout the auditory system of non-human mammals, from the AN (Miller and Sachs, 1983; Sinex and Geisler, 1983; Carney and Geisler, 1986; Sinex and McDonald, 1988, 1989), through the auditory midbrain (Chen et al., 1996; Chen and Sinex, 1999; Sinex and Chen, 2000) to at least the primary auditory cortex (AI) (Steinschneider et al., 1994, 1995, 2003; Eggermont, 1995; Aizawa and Eggermont, 2006).

This research, coupled with animal psychoacoustic experiments on speech discrimination in non-human mammals (Burdick and Miller, 1975; Kuhl, 1981; Hienz and Brady, 1988; Hienz et al., 1996; Engineer et al., 2008; Bizley et al., 2013), hints that at least some aspects of human speech perception are rooted in generalized auditory processing principles. However, this acoustically-driven approach is not well suited to more complex sets of speech sounds, since it is often difficult to define specific acoustic cues that underlie all phoneme contrasts. This is particularly the case in the context of natural variation in speech sounds, such as that between talkers (Nusbaum and Morin, 1992).

To address this, several more recent studies have taken a classifier-based approach in which the acoustic features that differentiate phonemes need not be explicitly defined *a priori* by the experimenter (Engineer et al., 2008; Mesgarani et al., 2008; Shetake et al., 2011; Perez et al., 2012; Centanni et al., 2013, 2014). Such studies typically focussed on the AI and are concerned with investigating patterns of neuronal activity that correlate with a behavioral outcome. However, a similar approach can be extended to the auditory system more broadly. Doing so makes it possible to investigate how the early auditory system addresses several key challenges in speech processing.

The first challenge faced by the auditory system is to robustly extract informative acoustic cues, such as those that discriminate phonemes, even in the presence of competing sounds. According to one hypothesis, this could be achieved by generating a sparse representation of the acoustic scene (Asari et al., 2006). The responses of neurons become increasingly diverse in the ascending auditory pathway as neurons appear to become more selective in their responses to complex spectro-temporal stimulus features and feature combinations (Sadagopan and Wang, 2009; Kozlov and Gentner, 2016). It has been suggested that this selectivity, and the resulting sparse representation may enhance discriminability of arbitrary sounds and thus provide a basis for robust phoneme recognition and language acquisition (Olshausen and Field, 2004; Hromádka et al., 2008; Mesgarani et al., 2008).

Secondly, whilst being sensitive to cues that differentiate phonemes, the auditory system must exhibit a degree of invariance to non-informative acoustic variation. For example, listeners need to recognize a word no matter who said it. The ability to recognize salient perceptual objects across physical variations in the stimulus is referred to as perceptual constancy (Kuhl, 1979; Summerfield, 1981) and is a phenomenon that is typically studied in the primary and higher-order areas of the AI in humans and other primates (Näätänen et al., 1997; Dehaene-Lambertz et al., 2002; Blakely et al., 2008; Molholm et al., 2014). However, it has been shown that neural activity in the AI of naïve ferrets is sufficient to encode phonemes despite variability across many talkers (Mesgarani et al., 2008). How and where in the brain such representations are produced is not well understood, although there is some evidence that the early auditory system, from cochlea to primary auditory cortex, plays an important role in developing invariant representations of natural sounds (Rabinowitz et al., 2013).

The neural representations involved in reconciling these challenges manifest spatially, in the distributed activity across a population, and temporally, in the sequence of action potentials produced by individual neurons. Sound is an inherently dynamic stimulus that modulates neural activity over time. However, it remains unclear at what timescale modulations in neural responses represent information necessary to identify phonemes. There is growing evidence that changes in neuronal activity over very short timescales (of the order of ∼1–10 ms) carries information about complex acoustic signals up to the level of the primary auditory cortex (Elhilali et al., 2004; Schnupp et al., 2006; Engineer et al., 2008; Wang et al., 2008; Huetz et al., 2009; Kayser et al., 2009, 2010; Panzeri et al., 2010; Garcia-Lazaro et al., 2013). However, it is not clear whether this temporal resolution is of value in real-world speech recognition.

To investigate how the auditory system addresses these challenges, we examined how the neuronal representation of a set of dynamic speech sounds changes throughout the ascending auditory pathway of naïve, anaesthetized guinea pigs. We obtained neuronal representations of these sounds from the AN using a computational model of the auditory periphery, and from the inferior colliculus (IC) and primary AI. To investigate the extent to which these representations facilitate invariance to natural acoustic variation, we obtained responses to multiple examples of each phoneme, produced by different talkers. We investigated how the information required to identify these speech sounds is represented within each of the brain regions using a neural classifier. We examined how this information is distributed across neural subpopulations and investigated the timescale of the neural code.

## Materials and Methods

### Subjects

Electrophysiological recordings were obtained from seven adult pigmented guinea pigs (*Cavia porcellus*). All procedures were carried out under the terms and conditions of licenses issued by the United Kingdom Home Office under the Animals (Scientific Procedures) Act 1986.

### Stimuli

The set of stimuli were chosen to match those used by Shannon et al. (1995), in which the authors were concerned with the perception of degraded speech, the neural bases of which were investigated in a set of experiments run in parallel to those presented here (unpublished). Since the speech recordings used in the aforementioned study were unavailable, a matching set of 16 vowel-consonant-vowel phoneme sequences (VCVs), each spoken by three male talkers with standard American Midwest dialect, were selected from the speech corpus recorded and described in detail by Shannon et al. (1999). Three male talkers were selected randomly from the full corpus to match the earlier study, which corresponded to talker IDs M2, M3, and M5 in the dataset obtained from the author. The medial consonants used were */b, d, f, g, k, l, m, n, p, s, ∫, t, ð, v, j*, and *z/*, which were in an */a/-*consonant-*/*a*/*context, where */*a*/* is an open back unrounded vowel as in “palm”. Again, this subset of the full 25 consonants described in the corpus was selected to match the earlier study.

As described in Shannon et al. (1999), these recordings were made in a double-walled sound-treated booth using a sample rate of 44.1 kHz and were stored in an uncompressed, 16-bit format. All recordings were band-limited to between 0.1 and 4 kHz to facilitate subsequent comparison to the parallel experiments on degraded speech sounds that were similarly band-limited as per Shannon et al. (1995). Each recording was aligned such that the medial consonant was approximately centered on the point 300 ms from stimulus onset. The recordings were then cropped to 700 ms in duration and a 10 ms raised cosine ramp was applied to both the onset and offset. The level of each stimulus was set such that the vowel portions had a mean intensity of 70 dB SPL and each stimulus was presented 10 times. In the electrophysiological experiments described below, stimuli were presented diotically via speakers (modified RadioShack 40–1377) coupled to hollow aural specula.

### Auditory Nerve Model

A computational model of the guinea pig auditory periphery was used to simulate AN representations of the stimulus set. The model takes a sound waveform as an input and calculates binary output sequences where a 1 symbolizes the occurrence of an action potential. The model has been described in detail previously (Sumner et al., 2002) and has been shown to reproduce responses to tones (Sumner et al., 2003a,b) and speech sounds (Holmes et al., 2004). In brief, the model comprises a linear filter approximation of the external auditory meatus, a dual-resonance non-linear filter bank model of the cochlea (Meddis et al., 2001), a biophysical model of transduction by the inner hair cell, stochastic spike generation and a model of adaptation based on quantal neurotransmitter dynamics at the synapse. Characteristic frequency (CF) is a parameter provided to the model. In this study, we simulated the responses of 100 fibers with CFs evenly spaced on a logarithmic scale from 0.1 to 5 kHz. Our implementation of the model is available online (Steadman, 2018).

### Electrophysiology

Animals were anaesthetized with urethane (1.3 g/kg in 20% solution, i.p.), supplemented as necessary to maintain suppression of the forepaw withdrawal reflex by 0.2 ml Hypnorm (fentanyl citrate 0.315 mg/ml, fluanisone 10 mg/ml, i.m.). Bronchial secretions were reduced with a premedication of atropine sulfate (6 μg/kg, s.c.). A tracheal cannula delivered 100% oxygen. Core body temperature was monitored and maintained at 38°C using a homeothermic blanket. Parts of both tragi were resected to expose the external auditory meatus and the condition of the tympanic membranes was checked for abnormalities. The animal was then secured in a stereotaxic frame inside a sound attenuating booth with the head secured in place with a bite bar and hollow aural specula through which acoustic stimuli were presented. Small holes were made in the auditory bullae into which long (0.5 mm diameter) polythene tubes were inserted to maintain pressure equalization.

Extracellular multi-unit recordings were made in the IC and primary AI using 16 channel multi-electrode arrays (NeuroNexus Technologies, Ann Arbor, MI, United States). Recordings were made in only the IC in two animals and only the AI in three animals. In the remaining two animals, recordings were made simultaneously in IC and AI. For IC recordings, a 5 mm by 5 mm craniotomy was made over the right IC (Rees and Palmer, 1988). For cortical recordings, the right temporalis muscle was resected such that the lateral suture was exposed, and the posterior portion of the orbit was visible. A craniotomy of approximately 5 mm by 5 mm was positioned such that it was approximately bisected by the lateral suture and the rostral edge was aligned with bregma (Wallace et al., 2000). In both cases, the dura under the craniotomy was resected and the exposed cortex was covered with warm agar (1.5% in saline).

Signals were digitized at 24.4 kHz, and multi-unit spikes were detected offline using custom software developed in MATLAB. Recordings were initially bandpass-filtered between 0.3 and 6 kHz using a zero-phase digital filter (fourth order, Butterworth). Robust signal statistics were used to determine a spike detection threshold, *T*, of four times the estimated standard deviation of the noise (Quiroga et al., 2004).

$$\begin{array}{c} T=-4\times median\;(\frac{\left|x\right|}{0.6745}) \end{array}$$

### Recording Site Characterisation

Pure-tone frequency response areas were acquired for each recording site. Tones of 50 ms duration, with 10 ms onset and offset raised cosine ramps, were presented diotically. Tone frequencies ranged from 0.2 to 25.6 kHz and increased in quarter-octave steps. Intensities ranged from 0 to 80 dB SPL in 5 dB steps. Tones were presented at a rate of five per second in randomized order. Firing rates in response to each tone were averaged across 10 repetitions and the CF was automatically extracted using the algorithm described in Palmer et al. (2013).

Two measures of sparseness were also calculated. The first was a measure of the degree of selectivity of neural responses to particular stimuli, referred to as the “lifetime sparseness.” The second was a measure of how activity is spread across a neural population, known as “population sparseness” (Willmore and Tolhurst, 2001). The metric for both measures was initially proposed by Rolls and Tovee (1995) and later refined to a normalized form (Vinje and Gallant, 2000). The calculation can be summarized as follows:

$$\begin{array}{c} S=\frac{1-{\left(\sum \frac{r_{i}}{n}\right)}^{2}/\sum \left(\frac{r_{i}^{2}}{n}\right)}{1-\frac{1}{n}} \end{array}$$

Where *r*_i_ is the average firing rate in the response to the *i^th^* stimulus in the case of lifetime sparseness, or the average firing rate measured at the *i^th^* electrode in response to a given stimulus in the case of population sparseness, and n is the total number of stimuli.

### Neural Classifier

To quantify the efficacy of the neural representations to recognize the speech stimuli, a nearest-neighbor classifier was developed in MATLAB. The design of this classifier was motivated by a need to provide as little prior knowledge to the classifier as possible and to include as few assumptions about complex downstream processing mechanisms. Initially, single trial peri-stimulus time histograms (PSTHs) of the first 650 ms of stimulus playback were produced using 1 ms bins. The final 50 ms were not used, since this was observed to correspond to the tail end of the final vowel portion, which was typically longer than the initial vowel. Thus, this response window better centered the response to the target, medial consonant stimulus.

These PSTHs were then smoothed by convolution with Hamming windows of 10 different lengths, evenly spaced on a logarithmic scale from 1 to 400 ms, where a length of one corresponds to no smoothing and a length of 400 yields representations that more closely reflect average firing rates. The analyses described below were carried out separately for every smoothing window duration. For population analyses, the single trial PSTHs of each simulated nerve fiber (AN) or multi-unit recording site (IC and AI) were concatenated to produce *M* × *N* matrices, henceforth referred to as neurograms, where *M* is the number of simulated fibers or recording sites and *N* is the number of time bins.

Subsequent processing was determined by which of three classifier modes was selected; *token, phoneme*, or *hierarchical*. The simplest was the *token* classifier mode, in which the classifier learned a separate class for each speech token (48 classes; 16 phonemes × 3 talkers). In this mode, neurograms corresponding to a single repetition of each stimulus were removed from the dataset before averaging all other neurograms across the remaining nine repetitions. Each of the removed single trial neurograms were then compared against the averaged neurograms in the manner described below.

The metric used to compare neurograms was Euclidean distance. If this were to be simply calculated using all spatio-temporal bins comprising the neurograms, this would effectively provide the classifier the absolute onset time of each stimulus, which the brain does not have access to. To avoid this, a time-shifting mechanism was implemented. For each comparison, the Euclidean distance was calculated for all relative lags from -100 to 100 ms in 1 ms steps. The test neurogram was classified as the token whose template it was nearest to for any given lag. Any bins outside the range of overlap were not included in the distance calculation by removing the first and last 100 bins (corresponding to 100 ms) from the templates. Distances were then given by:

$$\begin{array}{c} D_{i}=\sqrt{\sum_{m\;=\;1}^{M}\sum_{n\;=\;1}^{N}{\left(X_{m,n}-x_{m,n\;+\;i}\right)}^{2}} \end{array}$$

**FIGURE 1:**
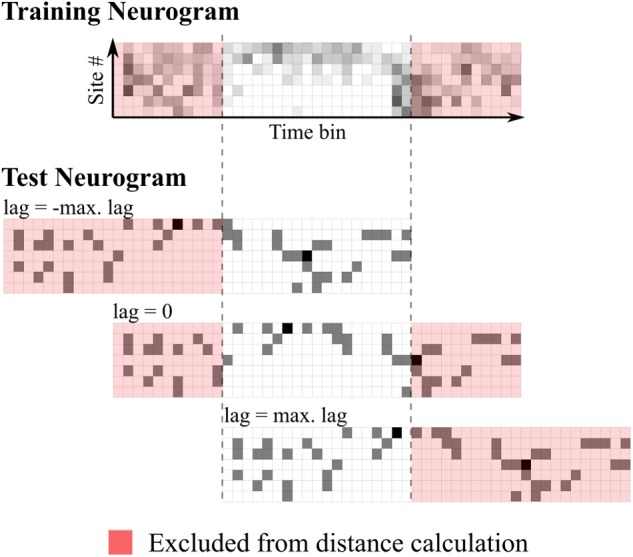
Schematic representation of time-shifting mechanism in the neural classifier. The top panel shows the training (template) neurogram for a given speech token. Below shows a single-trial test neurogram at three different lags. Euclidean distances are calculated using all unshaded bins.

Where *X* is a template neurogram, *x* is the test neurogram, *M* is the number of recording sites, *N* is the number of bins used in the distance measurement, equal to the length of the stimulus in milliseconds minus the maximum lag (100 ms) and *i* is a value ranging from 0 to 2 times the maximum lag. This mechanism is illustrated in **Figure 1**.

In the *phoneme* classifier mode, average neurograms in the training set were combined across talkers such that the classifier learned a single class per phoneme. Instead of simply averaging the neurograms across talkers, a time-shifting mechanism similar to that described above was used to minimize the pairwise distances between the average neurograms from individual talkers before averaging over all. Classification then proceeded in the same way as the *token* classifier. The *hierarchical* classifier operated in much the same way as the *token* classifier, but confusions between the same phonemes produced by different talkers were disregarded. This mode assumes a subsequent, simple processing stage that performs phoneme recognition by mapping distinct token classes to phoneme classes. The implementation of this classifier, along with an example script demonstrating its use is available online (Steadman, 2018).

### Discrimination Specificity

Similar percent correct values could be generated by a classifier that recognizes a single stimulus well, but is unable to distinguish between all others, to one that recognizes all stimuli, but with reduced precision. These two cases describe the distinction between *specialist* neurons, which can identify only a single stimulus but provide little information to discriminate between others, and *generalist* ones. To differentiate these two cases, we calculated a sensitivity index (*d*′; Macmillan and Creelman, 2004) for each stimulus using the following:

$$\begin{array}{c} d^{\prime }=Z(P_{HIT})-Z(P_{FA}) \end{array}$$

Where *Z*(*P_HIT_*) and *Z*(*P_FA_*) are the *z*-scores corresponding to the hit and false alarm rates, respectively, which were extracted from classifier confusion matrices. We then substituted *d*′ for *r* in the sparseness equation described above to obtain a normalized metric describing the shape of the distribution of *d*′ values. Values close to 0 correspond to a flat distribution indicating generalized performance and values close to 1 indicate specialist performance. We call this metric discrimination specificity. This measure has a key advantage over raw percentage correct, which is that it takes account of any response bias – i.e., propensity of the classifier to preferentially choose one consonant, regardless of which is correct.

## Results

### Representations of Speech

Neuronal representations of a set of natural speech sounds (16 vowel-consonant-vowel sequences, e.g., */apa/, /ata/, /ama/*, each produced by three talkers) were obtained from three stages of the auditory pathway: the AN, IC, and AI. A computational model of the peripheral auditory system was used to simulate the responses of 100 AN fibers. Multi-unit extracellular neural responses were recorded in 114 sites in the IC and 208 in the AI of anaesthetized guinea pigs. The complete dataset comprising spike times along with the stimuli is available online (Steadman and Sumner, 2018).

**FIGURE 2:**
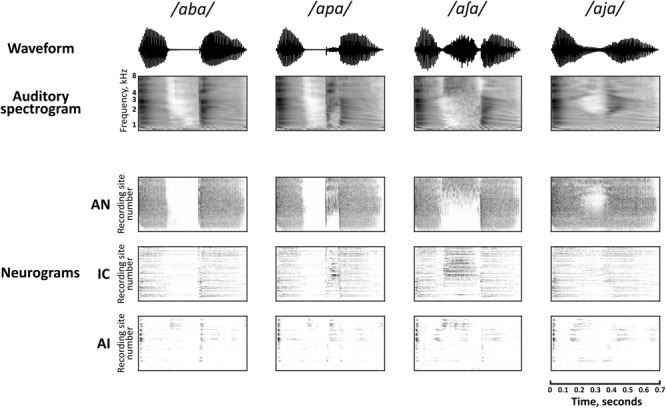
Auditory spectrographic representations of a subset of the speech tokens, */aba/, /apa/, /a∫ a/*, and */aja/* with the population responses to the same stimuli in the auditory nerve (AN), inferior colliculus (IC), and primary auditory cortex (AI). Auditory spectrograms were produced using the Matlab Auditory Modeling Toolbox (Søndergaard and Majdak, 2013) function audspecgram, whereby the frequency axis is displayed on an ERB-scale and the output is low-pass modulation filtered to reflect the spectrotemporal resolution of the cochlea. Neural population responses are shown as neurograms, whereby each row represents the spiking activity of a simulated nerve fiber (AN) or multi-unit recording site (IC, AI) using 10 ms bins, which are arranged by their characteristic frequency (CF) from low to high. Dark regions indicate higher firing activity. Note that this vertical scale does not match directly to the frequency scale of the spectrograms, since CFs are not uniformly distributed.

Spectrogram representations of four of the speech sounds are shown in **Figure 2**, along with the corresponding neural population representations in each of the brain regions. The neurogram representations of population responses comprise spiking activity in each of the simulated nerve fibers (AN) and multi-unit recording sites (IC and AI), arranged by their pure-tone CF. Frequency-specific neural responses are apparent in each of the nuclei. For example, simulated nerve fibers (AN) and multi-unit recording sites (IC and AI) with higher CFs show more activity than those with low CFs during the medial portion of the stimulus */a∫ a/*. It should be noted that the vertical scales of the neurograms and the spectrograms cannot be directly compared since CFs do not cover exactly the same range as the auditory spectrogram and are not uniformly distributed in IC and AI (however, the relevance of CF in the neural representation of these speech sounds across the three brain regions is discussed explicitly in section “Factors Influencing Speech Discrimination by Individual Units”).

There is a clear qualitative change in the representations as they are transformed via intermediate synapses in the ascending auditory pathway. Firstly, neuronal spiking activity is increasingly sparse at each downstream nucleus. In stark contrast to the activity of the simulated AN, cortical representations are largely transient with some exceptions (for example, ongoing activity can be seen in the high frequency region on AI in response to the fricative consonant */∫ /*).

The mean correlation between PSTHs in response to each of the 16 stimuli between sites with CFs within half an octave was calculated using 1 ms bin widths. The mean correlations were ρ_AN_ = 0.313, ρ_IC_ = 0.118, and ρ_AI_ = 0.128. A one-way ANOVA indicated a significant effect of brain region, *F*(2,258) = 39.3, *p* < 0.001. A Tukey *post hoc* test revealed that this reflected significantly lower correlations in IC and AI compared to AN (*p* < 0.001 in both cases). There was no significant difference between pairwise correlations in IC and AI (*p* = 0.932). This analysis indicates that recording sites with nearby CFs have similar response characteristics in AN, but are more heterogeneous in IC and AI, as suggested by the horizontal striations in the neurograms from these brain regions.

### Discrimination of Speech Sounds by Individual Units

The speech tokens elicited visually distinct patterns of neural activity in each brain region when averaged across multiple repetitions. However, in the real world the brain must perform speech recognition using only single-trial activity patterns. To reflect this, we used a template-based neural classifier to quantify how distinct these neural activity patterns were. To investigate the extent to which representations exhibit invariance within each phoneme class, the classifier was operated in three different modes, *token, phoneme*, and *hierarchical* (see “Materials and Methods”).

For representations that exhibit invariance across talkers it is expected that classification of individual acoustic waveforms (*token* classification) would result in confusions within each phoneme class. For example, it would be difficult to discriminate */aba/* produced by talker one from the same VCV produced by talkers two and three. In this case, *phoneme* classification, which is designed to classify on the basis of medial consonant identity, would perform significantly better. *Hierarchical* classification utilized an initial stage identical to token classification, but confusions between the same phoneme produced by different talkers were ignored. This mode predicts performance with the assumption of a subsequent, simple processing stage that performs phoneme recognition by mapping distinct token classes to phoneme classes.

**FIGURE 3:**
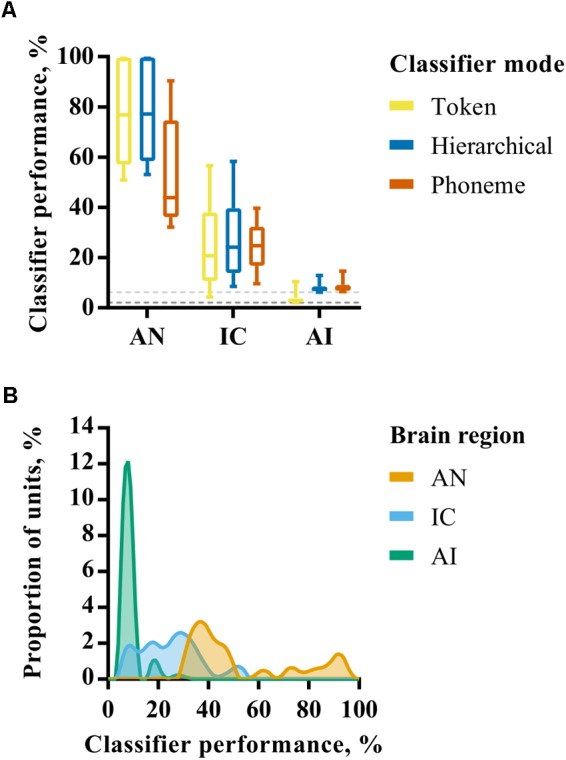
**(A)** Discriminability of simulated auditory nerve fiber (AN) and multiunit responses in the IC and AI for each of the three classifier modes. The values shown correspond to classifier performance using the optimal smoothing window (1–400 ms) for each unit. **(B)** Smoothed histograms showing the distribution of classifier performances for each of the three brain regions using only the phoneme classifier mode.

The performance of each neural classifier mode for individual recording site representations in each brain region is summarized in **Figure 3A**. These data reflect the maximum classifier performance for any smoothing window length; the effect of smoothing is explicitly addressed subsequently. Given the bounded nature of classifier output values (between 0 and 100%) and the non-normal distribution of percent correct values within each brain region (**Figure 3B**), nonparametric statistics were used to analyse these data. A Kruskal–Wallis H test revealed a statistically significant difference in token classifier performance between the three brain regions, *χ*^2^ = 302, *p* < 0.001, with mean rank scores of 366.5, 250.7, and 115.5 in AN, IC, and AI, respectively. A Jonckheere–Terpstra test for ordered alternatives confirmed that there was a statistically significant trend of lower classifier performance within higher auditory nuclei, *J* = 2878, *z* = 18.8, *p* < 0.001. The same analysis was carried out for each classifier mode, which produced similar results with *p* < 0.001 in all cases.

These analyses reveal a progression from highly redundant, rich representations in the AN in which individual fibers alone can convey sufficient information to recognize speech sounds when presented in quiet, to one in which the activity of a small number of neurons in the IC and AI is insufficient to perform the recognition task with a high degree of precision. Indeed, the classifiers performed little better than chance, on average, when provided with multi-unit activity from individual recording sites in AI. This change is progressive, with the IC occupying a middle-ground between AN and AI.

As described above, differences between classifier modes can be used to investigate the extent to which representations exhibit invariance. In the AN, the hierarchical classifier performs similarly to the token classifier, suggesting that the confusions made by the classifier are not predominantly across talkers (i.e., within phoneme classes) as would be expected for representations exhibiting across-talker invariance. The phoneme classifier also performs significantly worse than the token classifier (Mann–Whitney U, *p* < 0.001). These results indicate that, whilst the AN is sensitive to acoustic features that identify specific speech tokens, the responses do not form a representation space in which speech tokens of the same phoneme class are more similar to one another than those of other phoneme classes.

In the IC there is no such penalty for using a phoneme rather than a token classifier. Both token and phoneme classifiers perform similarly (Mann–Whitney U, *p* = 0.39). One explanation for this could be that these representations appear to be more robust to across-talker differences. However, it should be noted that the hierarchical classifier performance is not markedly higher than the token classifier and this is in the context of lower overall classifier performance. In the AI the phoneme and hierarchical classifiers both perform better than the token classifier, but performance is close to chance in both cases. Since the token classifier has more classes than the hierarchical and phoneme classifiers (48 tokens vs 16 phonemes), this is expected and not necessarily indicative of greater invariance.

To summarize, these analyses reveal a progression from highly redundant, rich representations in the AN in which almost any fiber contains sufficient information to discriminate between a large set of speech sounds, to one in which the activity of a small number of neurons in the IC and AI is insufficient to perform the discrimination task with a high degree of precision. This change appears to be progressive, with the discriminability of responses in the IC occupying a middle-ground between AN and AI. Furthermore, at the level of individual units, we find little evidence of increasing invariance within phoneme classes in the ascending auditory pathway.

### Factors Influencing Speech Discrimination by Individual Units

Within each brain region, there is a large amount of variability in classifier performance using individual nerve fiber or multi-unit representations. For example, the token classifier was able to identify the correct speech token 100% of the time using responses from single simulated AN fibers (as long as they have an appropriate CF). Using another fiber, however, resulted in classifier performance dropping to only 22.7%. Similarly, classifier performance ranged from approximately chance (2.08%) to 82.1% when trained and tested using multi-unit responses in the IC. To understand this variability, we investigated the relationship between various classical descriptors of neuronal spiking behavior and classifier performance.

**FIGURE 4:**
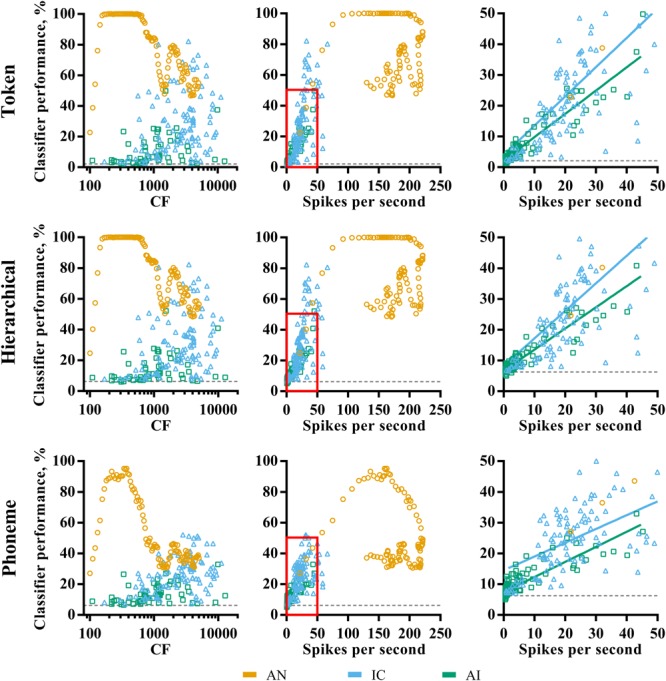
Characteristic frequency (left column) and total average firing rate (middle column) as a function of classifier performance for each brain region (color; see legend) and classifier mode (row). The right column shows a magnified view of the region close to the origin on the firing rate vs. classifier performance axes, and the line of best fit for IC and AI. Sites that did not demonstrate frequency specific tuning to pure-tones in IC and AI are omitted. All points indicate the discriminability using the optimal smoothing window for each recording site.

**Figure 4** shows the distribution of pure-tone CF and mean evoked spike rates, and their relationship to classifier performance in each of the three brain regions. The AN fibers appear to fall into two groups; those with highly discriminable (∼100% correct) responses and those for which classifier performance falls below 80% for the token and hierarchical classifier modes, with few data points between. As shown in **Figure 3A**, AN classifier performance is generally lower when using the phoneme classifier but follows a similar pattern.

Inspection of the relationship between fiber CF and discriminability reveals that these groups correspond to fibers with low and high CFs. The cut-off is at around 1 kHz, which is notably similar to the point at which phase locking deteriorates in the AN of the guinea pig (Palmer and Russell, 1986). We note that this is also the reciprocal of the bin size used in the creating of the PSTHs (1 ms). To check that this was not an artifact of binning the responses, the same analysis was performed using a bin size of 0.1 ms, which resulted in very similar results and did not shift this cut-off to higher frequencies.

The relationship between CF and classifier performance in IC and AI is more linear, with higher CFs corresponding to higher performance. This is even true for those neurons tuned to well above 4 kHz, where stimulus energy is attenuated (see “Materials and Methods”), suggesting the correspondence between the pure-tone tuning and the energy in the stimulus is a poor predictor of how efficacious neurons were for classification. A Pearson product-moment correlation analysis revealed that this relationship is strongest for the phoneme classifier mode and was significant in IC, *r* = 0.36, *p* < 0.001, but not in AI, *r* = 0.23, *p* = 0.08. With respect to mean firing rate, the AN data fall into the two groups described previously. In IC and AI, mean firing rate was significantly correlated with classifier performance regardless of classifier mode (right panels of **Figure 4**), an effect that was strongest for the token classifier, ρ_IC_ = 0.68, ρ_AI_ = 0.94, *p* < 0.001 (in both cases).

These analyses suggest that the ability of an individual AN fiber to identify speech sounds is broadly determined by stimulus acoustics and the fiber CF. High firing rates do not necessarily indicate greater classifier performance. In the IC and AI, however, CF is a poor indicator of classifier performance, whereas firing rate is.

**FIGURE 5:**
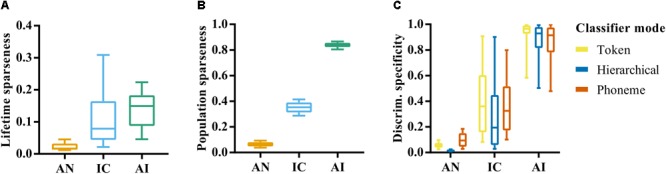
**(A)** Lifetime sparseness of individual site responses in the auditory nerve, inferior colliculus, and auditory cortex. Values close to 0 indicate a tendency to respond to each stimulus with equal firing rates. Value closer to 1 indicate a tendency to respond selectively to a small number of speech tokens. **(B)** Population sparseness of responses in AN, IC, and AI. Values close to zero indicate that the neurons across the population respond similarly to a given stimulus. Values closer to 1 indicate that stimuli elicit responses from a small subset of the population. **(C)** Discrimination specificity in the AN, IC, and AI for each classifier mode. Discrimination specificity is based on a commonly used measure of sparseness applied to d’ values calculated from confusion matrices. Values close to 0 indicate an ability to discriminate all tokens equally. Values close to 1 indicate an ability to distinguish only a small subset of stimuli.

The relationships between CF, firing rate and classifier performance appear to be quite similar in IC and AI, as does the overall distribution of these attributes, but it is possible that differences between these brain regions emerge in when and where these neurons tend to fire. A measure that is related to firing rate, but provides more information about the selectivity of responses, is *sparseness*. Neural responses are said to exhibit lifetime sparseness if responses are highly selective to a small number of stimuli. **Figure 5A** shows the lifetime sparseness of the neurons in our sample in each of the three nuclei. The mean sparseness values increased in the ascending nuclei (*μ* = 0.03, 0.12, and 0.14, respectively). A one-way ANOVA was conducted to test the effect of brain region on lifetime sparseness, which confirmed a significant effect, *F*(2,419) = 68.8, *p* < 0.001. *Post-hoc* comparisons using the Tukey HSD test indicated that the mean lifetime sparseness in the AN was significantly different to that in the IC and AI. The difference in mean sparseness between the IC and AI was not significant, *p* = 0.052.

**Figure 5B** shows a second measure of sparseness: the population sparseness, and how this varies across each brain region. This measure is an indication of the tendency of neurons across a population to respond with a similar strength, with values close to one indicating that only a small subset of neurons responds to any given stimulus. In this case the error bars reflect variation across stimulus repetitions rather than across individual recording sites, given that it relates to the population response. A one-way ANOVA revealed a significant effect of brain region on population sparseness, *F*(2,141) = 7408.16, *p* < 0.001. This reflects an increase in population sparseness from AN to AI; a further indication of increasing heterogeneity of responses throughout the ascending auditory system.

These analyses highlight the dense and redundant nature of representations in the AN given the very low values of sparseness and corresponding high classifier performance. Differences between IC and AI emerge when the way activity is distributed across the neural population is considered, which could suggest a more distributed representation in AI compared to IC. This could explain the much lower discriminability values seen in AI on average and supports the idea that neurons in higher auditory nuclei become more selective to features of complex auditory stimuli.

We hypothesized that this higher selectivity would result in specialized neurons that are able to identify a single speech token, but are unable, in isolation, to discriminate between a larger set of stimuli. On the other hand, the generalist neurons in the more peripheral nuclei would discriminate between all speech tokens equally well. To measure this, we developed a normalized measure of discrimination specificity, which approaches 0 for neurons with a similar recognition accuracy for each of the speech tokens and 1 for those that tend to only recognize one single speech token much better than all others (see “Materials and Methods”).

**Figure 5C** shows that mean discrimination specificity does indeed increase in the ascending auditory nuclei. A one-way ANOVA confirmed a significant effect of brain region on neural discrimination specificity, *F*(2,419) = 836.50, *p* < 0.001, and *post-hoc* analysis revealed significant differences between the AN and IC, *p* < 0.001, as well as between the IC and AI, *p* < 0.001. It also demonstrates the broad distribution of discrimination specificity scores by neurons in the IC, suggesting a continuum of functionality from generalist to specialist.

**FIGURE 6:**
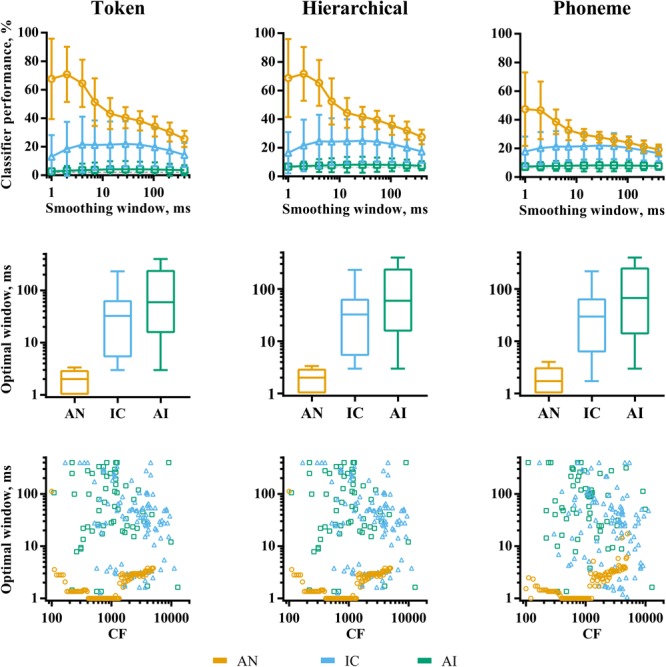
The effects of smoothing window length on discriminability of responses to VCVs by individual recording sites in AN (orange), IC (blue), and AI (green) for the three classifier modes. The top row shows the effect of smoothing averaged across recording sites within each brain region. The middle row shows the distribution of optimal smoothing windows. The bottom row shows the relationship between characteristic frequency and optimal smoothing window.

The analyses so far have considered classifier performance when responses were smoothed using optimal temporal smoothing windows, which were found by parametrically varying the window lengths. **Figure 6** shows a summary of the effects of smoothing on classifier performance for each brain region. The effects are very similar, regardless of classifier mode. The top row of the figure shows classifier performance as a function of smoothing averaged across recording sites. For the AN data, there is a clear peak in classifier performance for very short smoothing windows, reflecting reliable, precisely timed spikes that differ across the speech tokens. This peak is greatly diminished for the phoneme classifier mode, suggesting that the representations based on precise spike timing encode acoustic features that do not generalize well across talkers, such as the temporal fine structure. Conversely, precise timing in the IC and AI does not improve classifier performance. Indeed, in the IC, a degree of smoothing appears to improve it.

Overall, our analyses of individual recording sites demonstrate an evolution of the neural code throughout the ascending auditory pathway. In the AN, each fiber contains rich information about all the speech stimuli presented, reflected by high classifier performance and low discrimination specificity. Here, acoustic information is encoded in many, precisely timed spikes, particularly within fibers whose CF falls well within the limit of phase-locking. As information progresses up the auditory pathway, responses become increasingly sparse, with localized neuronal activity encoding a smaller range of stimulus features, indicated by the progressive increase in both lifetime sparseness (within recording sites) and population sparseness (across neuronal populations), resulting in low overall classifier performance and high discrimination specificity. Optimal smoothing windows in the higher auditory nuclei are also longer, with precise spike timing being less useful for speech recognition than spike rates over 10–100 ms epochs.

The classifier modes had a significant effect on classifier performance in general. This was most pronounced in the AN where the phoneme classifier, in which templates were combined across multiple talkers, performed significantly worse than the token classifier. The hierarchical classifier did not perform markedly better than the token classifier in any brain region, which indicates that there is little evidence of representations facilitating invariance to across-talker differences.

There are two possibilities that may explain the lower overall classifier performance in the IC and AI compared to the AN. One is that the information necessary to perform the consonant discrimination task is not maintained across multiple synapses in the ascending auditory system. Another is that this information is encoded in spiking activity distributed across neuronal populations. Subsequent analyses will aim to address which is the case by providing the classifier with activity from neuronal populations, rather than individual recording sites.

### Discriminability of Population Responses

We have demonstrated that single AN fiber representations of speech tokens produced by multiple talkers can be reliably discriminated by a template-matching classifier. The same cannot be said of multi-unit neuronal representations in the IC and AI. We investigated how the discriminability of these representations was affected by training the classifier using representations comprising larger populations of neurons. The classifier methods are identical, except that classification is performed using spatio-temporal population representations instead of spike trains produced by single nerve fibers or recording sites.

**FIGURE 7:**
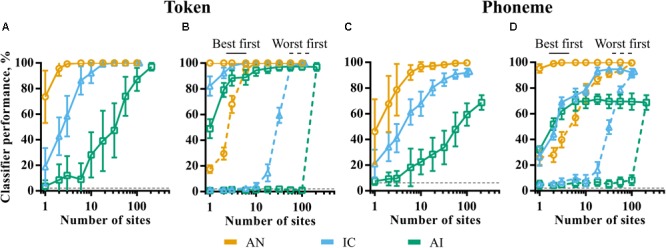
The effect of population size on classifier performance for token **(A,B)** and phoneme **(C,D)** classifier modes. In panels **A,C**, 20 subpopulations were selected randomly within each brain region. Error bars represent variation across these subpopulations. In panels **B,D**, sites were selected in descending (best first) and ascending (worst first) order of discriminability corresponding to the solid and dashed lines, respectively. The smoothing windows that yielded maximum performance for the whole population for each brain region were used.

**Figure 7A** shows token classifier performance as a function of the (randomly sampled) population size in each of the brain regions. Despite the generally poor ability of individual recording sites in IC and AI to discriminate the speech tokens, perfect classifier performance is possible by combining the responses across relatively small neuronal populations. The required population size increases by orders of magnitude from AN to IC to AI. In the AN, more than three fibers guaranteed perfect discrimination. Twenty or more IC units are required to match this, whilst the total recorded population of 208 multi-unit sites is required in the AI.

These results could be indicative of the following scenarios. Firstly, this may indicate that the information driving classifier performance becomes increasingly distributed across the population, with each neuron encoding sufficient information to distinguish only a few speech tokens from all others. This might be the case if, for example, a particular neuron was sufficiently selective so as to only spike in response to one of the speech tokens. Alternatively, it may be that much of the information driving the speech token discrimination task is encoded in the activity of a small number of generalist neurons, able to discriminate the majority of the tokens from one another. In this case, increasing the size of the subpopulation increases the chances that one or more of these high-performing generalist neurons are included.

In order to distinguish these two scenarios, we sampled neuronal subpopulations in order of token classifier performance measured individually. The results are shown in the solid and dashed lines of **Figure 7B**. The solid lines are the results where only the best *N* sites are selected. Conversely, the dashed lines correspond to the same analysis selecting the worst *N* sites. These data are indicative of the latter scenario; classifier performance reaches a maximum with very small, highly informative populations in all the nuclei with much of the population contributing very little information. These sites are poorly predicted by CF. Indeed, they appear to have pure-tone CFs significantly higher than the low frequencies containing most of the energy in the speech stimuli and are generally distinguished by their higher average firing rate, as shown previously in **Figure 4**.

The results of the same analysis using the phoneme classifier, in which templates incorporate across-talker variability, are shown in **Figures 7C,D**. A comparison of **Figure 7C** and **Figure 7A** shows that, for a given population size, when not at ceiling, performance is generally worse in all nuclei. For representations that become less sensitive to the fine-grained differences between the same phonemes produced by different talkers, it would be expected that the token classifier would make confusions between exemplars of the same phoneme. In this case, the phoneme classifier would perform better, which is not the case; both at the neuronal and population level, we see little to suggest that these representations could facilitate invariance to across-talker differences.

**Figure 7D** shows that, similar to the token classifier results in **Figure 7B**, the majority of information driving classifier performance is encoded by small, optimal populations. IC and AI performance initially grows at a similar rate (blue and green solid lines). This contrasts with the corresponding token classifier data (**Figure 7B**), where IC performance is higher for very small populations. This implies that the best performing units in AI do exhibit at least some greater degree of generalization within phoneme classes than in the IC.

**FIGURE 8:**
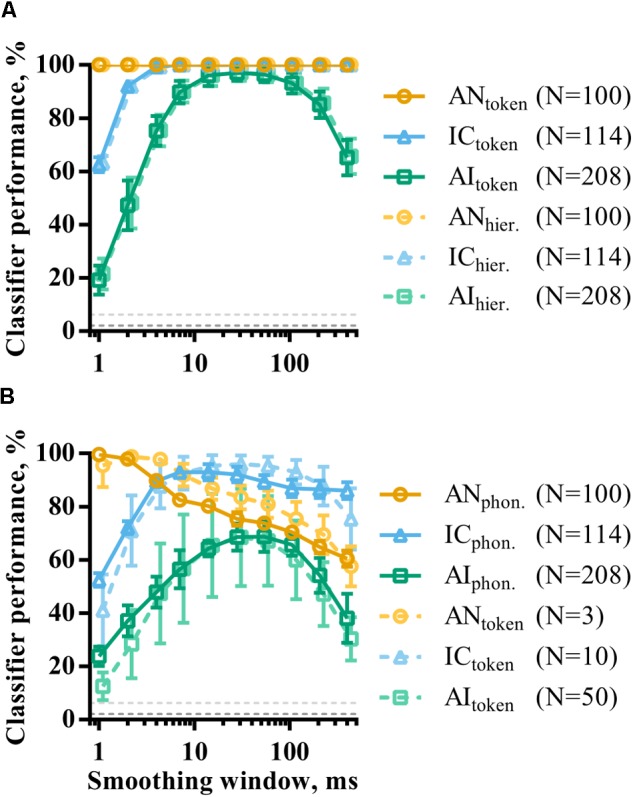
The effect of smoothing on the discriminability of population representations of VCVs using token **(A)** and phoneme **(B)** classifier modes. Also shown is the same analysis for hierarchical classifier mode (dashed line, **A**). The dashed line in **B** is data from the token classifier using neuronal subpopulations from each brain region. The sizes of these subpopulations are indicated in the figure legend and were chosen such that the maximum token classifier performance was matched to that of the phoneme classifier.

The effect of population size on consonant discrimination described above considered classifier performance using optimal smoothing windows. As in the analysis of single recording site responses, population responses were also temporally smoothed by convolution with a window of parametrically varied duration. **Figure 8A** shows how token classifier performance varies as a function of window length. AN and IC representations are evidently highly redundant for speech token recognition; even representations using a 400 ms smoothing window are sufficient for perfect classifier performance. This is in contrast with the results of the same analysis applied to single recording sites (**Figure 6**) and indicates a viable rate-place code for consonant recognition (at least when those consonants are presented in quiet at the sound level used in this study).

In AI, the shortest smoothing windows result in very poor classifier performance, indicating that the millisecond-precise timing of spikes in responses to the target sounds is unreliable across multiple presentations. Instead, a degree of smoothing improves discriminability. However, classifier performance is also reduced by using the longest smoothing windows, showing that the temporal structure of the neuronal response must be considered. Cortical neuronal representations with dynamics over timescales of between 10 and 100 ms appear to maximize discriminability.

**Figure 8B** shows the same analysis using the phoneme classifier mode. In all three brain regions, the discriminability of the neural representations generally decreased, as shown previously. This effect was most prominent in AI. Temporal smoothing had a dramatically different effect on discriminability in the AN and IC; increasing smoothing window durations caused a corresponding decrease in discriminability in AN. Discriminability of IC representations, on the other hand, is far more robust to severe temporal degradation of the spike trains, such that providing only the spatial distribution of firing rates as an input to the classifier still resulted in over 80% correct. For AI representations, the function shows a more clearly defined optimal timescale, with a best smoothing window of 54 ms.

The results from the phoneme classifier show clear shift toward longer optimal timescales from AN to AI. However, it is difficult to compare this to token classifier where performance is at or near ceiling in many cases. To facilitate this comparison, the token classifier was run using subpopulations that resulted in similar maximum classifier performance to the phoneme classifier. This corresponded to subpopulations of *N* = 3, *N* = 10, and *N* = 50 in AN, IC, and AI, respectively, as indicated in the figure legend. The results of this are shown in the dashed lines of **Figure 8B**. The resulting curves are remarkably similar regardless of classifier mode.

In summary, the reduced ability of neuronal activity to discriminate between consonants in the IC and AI compared to the periphery is largely recovered by considering population activity. However, this code appears to be confined to small subpopulation of particularly informative neuronal subpopulations. In the AN, and to a lesser extent the IC, the coding of speech tokens, as opposed to phonemes, is highly redundant; putative neural codes on multiple timescales provide sufficient information. Optimal timescales become longer in higher auditory nuclei.

## Discussion

We obtained responses to an extensive set of consonants at multiple stages of the central auditory system. Reponses in the AN were simulated using a computational model. Extracellular multi-unit activity was recorded in IC and AI of naïve, anaesthetized guinea pigs. The neuronal representation of these sounds was transformed from a dense and redundant one in the auditory periphery to a spatially and temporally sparse one in the AI. Nonetheless, these representations encode sufficient information to discriminate an extensive set of speech sounds in a naïve, anaesthetized animal model. The optimal timescale of neuronal activity facilitating recognition of the dynamic speech sounds used in this study increased from a few milliseconds in the periphery to several tens of milliseconds in the cortex, with little extra salient information gained by considering timing over shorter epochs. However, there is little evidence that these longer timescales are associated with increased invariance across talkers; rather, it seems likely that the auditory system up to the level of the primary cortex performs a dimensionality expansion, which could putatively form the bases of flexible and robust complex sound recognition by downstream cortical regions.

Our results are very much in line with previous literature showing that key acoustic cues for consonant discrimination, such as the voice onset time (VOT; Sinex and McDonald, 1988, 1989; Steinschneider et al., 1994, 1995, 2003, 2013; Eggermont, 1995) are represented throughout the auditory system of non-human mammals, and that such representations are sufficient to discriminate phonemes (Engineer et al., 2008; Shetake et al., 2011; Perez et al., 2012; Centanni et al., 2013, 2014).

Previous work using a classifier approach has focused primarily on the cortex, and direct comparisons of neuronal and behavioral speech token discriminability within the same species. We were interested in how information is propagated and transformed throughout the auditory system, and how some of the speech recognition challenges faced by listeners influence the value of various putative neural codes. We therefore modified the classifier approach and extended it to three key stages in the auditory processing pathway; the AN, IC, and AI.

It has been suggested that the neuronal representation of speech evolves throughout the ascending auditory system from one that is primarily driven by stimulus acoustics to one that more closely reflects perception (Perez et al., 2012). Our experiment was designed to investigate the nature of this transformation and its implications in the neural coding of speech, whilst considering some of the challenges in real-world speech recognition. Firstly, we used a stimulus set incorporating natural variability by using multiple talkers. This conceptually changes the nature of the neural classifier paradigm, as well as the analogous behavioral task in an important way. Imagine, for example, we were interested in testing a subject’s ability to discriminate images of apples vs oranges. If a subject is only shown one image of an apple and one of an orange, the subject need not necessarily utilize cues that generalize to other images of the fruits, such as the color. Many other parameters specific only to the images used can define the distinction, such as overall brightness, to give one example.

Secondly, we focussed on representations of an extensive set of word-medial consonant sounds (*/a/-*consonant-*/a/*). This is significant, since it is likely that neuronal responses to word-initial consonants, may be different in nature from those to consonants in continuous speech due to an apparent temporal asymmetry in auditory perception; listeners appear to be much more sensitive to spectral and temporal features of sound onsets compared to offsets (Phillips et al., 2002).

Finally, we introduce a time-shifting mechanism in our classifier, such that it is not provided with the absolute stimulus onset time. As has been pointed out previously (Centanni et al., 2014), this is an important consideration as the brain does not have access to this external information. Our time-shifting approach differs from previous ones as it only depends on spiking neural activity of the population under consideration and requires no baseline measure derived from the activity of a broader population.

The finding that cortical responses to complex sounds are typically transient and sparse, even in awake subjects, is not new (e.g., Hromádka et al., 2008). However, here we show that such responses constitute a distributed representation of the necessary cues to reliably discriminate an extensive set of speech sounds. Moreover, we find this to be the case in the central auditory system of a naïve, anaesthetized animal model. Furthermore, we show that the transformation appears to be progressive, with representations in the auditory midbrain exhibiting intermediate characteristics, between the AN and primary cortex.

These findings are also consistent with existing literature on how the representation of complex sounds in general evolve along the ascending auditory pathway. For example, Chechik et al. (2006) recorded responses of auditory neurons to birdsong in the IC, medial geniculate body (MGB) and AI of anaesthetized cats. They found that the amount of information conveyed by individual neurons was significantly lower in the AI and MGB compared to the IC, whilst representations across small neural populations were less redundant. This is also consistent with the sparse coding hypothesis, whereby sensory systems generate increasingly efficient representations of natural sounds (Olshausen and Field, 2004; Hromádka et al., 2008; Mesgarani et al., 2008).

The development of representations that are invariant to fine-grained, non-informative acoustic variation implies a decrease in selectivity to complex acoustic features, not the increase that we observe. Indeed, we find little evidence that representations in the ascending auditory system facilitate invariance. Our findings support an alternative model, which posits that the role of the auditory system up to at least the primary cortex is to create a multidimensional representation that forms that basis of flexible and robust class boundary definitions later on (Olshausen and Field, 2004; Hromádka et al., 2008; Mesgarani et al., 2008). It seems likely that this is achieved in higher auditory neurons by developing sensitivity to nonlinear combinations of spectrotemporal features, as has been observed in songbirds (Kozlov and Gentner, 2016) and awake marmosets (Sadagopan and Wang, 2009). Such a representation could facilitate complex tasks such as listening in noise, since signals that become degraded across one acoustic dimension (through energetic masking, for example) could remain intact in others.

Human phoneme perception is clearly affected by years of experience and becomes specialized for an individual’s linguistic environment. This is evident in the way that human infants can be sensitive to non-native phonemic contrasts in a way that adult listeners are not (for a review, see Kuhl, 2004). Nonetheless, intracranial recordings in humans suggest that responses to speech appear to be driven primarily by stimulus acoustics rather than phonetics (Mesgarani et al., 2014). This provides further support to the idea that language development is guided by auditory processing mechanisms shared across mammalian species and suggests that the organizing principle described above might be preserved in humans. If this is the case, changes in speech perception resulting from auditory experience is likely to depend largely on connectivity between primary and downstream cortical regions and subsequent neural processing.

We investigated the efficacy of neural codes at various timescales for discriminating between phonemes. The optimal timescales increase from milliseconds in the periphery to tens of milliseconds in the AI. Representations comprising neural codes on a millisecond timescale lead to classifier performance significantly above chance in all brain regions, which demonstrates that this does encode stimulus information, but it is not required, nor is it optimal in the two higher brain regions in this study. Indeed, little benefit is gained by providing a neural classifier with response dynamics on timescales shorter than around 100 ms in the AI.

This appears to contrast with several recent classifier-based studies that have suggested that millisecond-precise timing in the primary cortex plays a role in speech discrimination. For example, Engineer et al. (2008) recorded responses to a set of consonants in the AI of rats and quantified their discriminability using a nearest-neighbor classifier. They reported that the ability of their neural classifier to discriminate consonant pairs better correlated with the rat’s ability to discriminate the sounds behaviourally when neuronal responses with a 1–10 ms timescale were used, compared to firing rates over the entire 700 ms duration of their consonant-vowel stimuli. Subsequent studies using a similar methodology have found comparable results for consonant stimuli (Shetake et al., 2011; Perez et al., 2012).

This apparent contradiction could be due to several key methodological differences. Firstly, with one notable exception in which timescales of the neural code were not explicitly examined (Mesgarani et al., 2008), the conclusions of these previous studies are limited to pairwise discrimination of speech tokens to facilitate comparison with a go/no-go behavioral task. It may be that in the context of a more comprehensive set of speech sounds millisecond-timescale cues are more ambiguous and therefore difficult to interpret. Furthermore, it may be that incorporating natural variability by using multiple talkers diminishes the salience of fine-grained acoustic cues and therefore the viability of a precise timing code for the analogous behavioral task (although it should be noted that we find comparable optimal timescales in our speech token compared to our phoneme discrimination task, so this is not a complete explanation).

Secondly, it appears that the precision of spike timing differs between onset responses and responses to ongoing sound (Phillips et al., 2002). In most of the previously mentioned studies, onset responses to word-initial consonants were examined. From these comparisons, it is clear that any conclusions about the role of putative neural codes for speech discrimination are profoundly affected by the stimulus paradigm. For example, optimal decoding of cortical responses to vowel sounds (Perez et al., 2012), guinea pig vocalizations (Huetz et al., 2009) and marmoset calls (Schnupp et al., 2006) appears to occur when timescales of tens of milliseconds are used.

There are several limitations on the extent to which the findings of this study may be generalized to the neural coding of speech in general. The first of which is that our stimuli were presented in the absence of background noise and at a single sound level. The effects of added noise on neural coding of complex sound have not been extensively studied, though one study demonstrated that longer temporal integration windows are used to discriminate pairs of speech sounds in noise relative to the same sounds presented in quiet in the AI of rats (Shetake et al., 2011). However, as mentioned previously this study focussed on the discrimination of pairs of speech sounds and it is unclear to what extent cortical rate encoding over long (50–100 ms) epochs is sufficient to recognize sounds from a more extensive corpus.

Another limitation is that all electrophysiological recordings were made in a urethane-anaesthetized model. The effects of this anaesthetic on neural coding in the mammalian peripheral auditory system remain poorly understood and to the best of the authors knowledge no direct comparison of awake and anaesthetized responses to complex sound in the AN has been reported. However, one study did find that several anaesthetic agents (not including urethane) did have differential and significant effects on thresholds, tuning and firing rates in the AN of the gecko (Dodd and Capranica, 1992).

Likewise, differences between neuronal representations of complex sound in awake and urethane-anaesthetized models in the IC have not been directly investigated, as far as the authors are aware. However, one report compared discriminability of neuronal responses to complex sound in the anaesthetized and awake avian auditory midbrain and found that, while intrinsic excitability was depressed, neural coding (as measured by spectral tuning properties and the discriminability of responses to natural birdsong) was not significantly affected (Schumacher et al., 2011).

The differences between awake and anaesthetized models have been more extensively reported in the AI. It is commonly reported, for example, that anaesthesia has the effect of suppressing ongoing responses to acoustic stimuli, leading to an overrepresentation of stimulus onset not apparent in the awake AI. This indeed appears to be the case with steady-state, or brief stimuli. However, sustained responses have been observed in the awake auditory cortex, provided that the stimulus is optimal for a given neuron (Wang et al., 2005). Here, we report many recording sites in the anaesthetized AI that also demonstrate sustained responses. This might reflect that we presented natural, complex stimuli which are more likely to contain spectro-temporal dynamics that overlap with the optimal stimulus space for any given neuron.

The tuning properties of cortical neurons also appear to be affected by anaesthesia, the effect of which is to produce a reduction in the proportion of neurons with complex, circumscribed spectro-temporal receptive fields (STRFs; Wang, 2018). An increase in the complexity of cortical STRFs would suggest that it is possible that auditory cues facilitating speech recognition could be distributed even more broadly across the population than we observe here. With respect to temporal dynamics, Ter-Mikaelian et al. (2007) showed that anaesthesia can increase the temporal precision of cortical neurons to synthetic stimuli. On a related note, non-synchronized rate-encoding of click trains have only been reported in awake animals (Lu et al., 2001; Dong et al., 2011; Gao and Wehr, 2015), with anaesthetized models exhibiting synchronized responses. However, it remains unclear how anaesthesia affects the temporal dynamics of responses to ongoing, natural, complex sound.

In future studies investigating the importance of spike timing in speech discrimination, it is important to draw a distinction between token and phoneme recognition paradigms. It will be informative to apply corresponding classification algorithms to distinguish auditory object (e.g., phoneme) vs. waveform (i.e., token) encoding. We did not find evidence of invariant phoneme encoding from AN to AI. However, it would be interesting to extend this approach beyond the primary AI, and to either use conspecific calls or sounds on which the animals have been trained. Future similar studies should also include natural variation by using multiple exemplars of each target sound and consider potential differences in neuronal encoding schemes emerging from acoustic context (e.g., is the target sound preceded by other sounds, or is it presented after a period of silence). Furthermore, signal processing tools can be used to systematically manipulate the spectro-temporal complexity of speech. Such tools will be useful to distinguish between true spike-timing based representations and rate codes that fluctuate with rapid changes in the stimulus (Theunissen and Miller, 1995).

## Author Contributions

CS and MS conceived of the study and developed the experimental design. MS collected all the experimental data, designed and implemented analysis software and wrote the manuscript with input from CS.

## Conflict of Interest Statement

The authors declare that the research was conducted in the absence of any commercial or financial relationships that could be construed as a potential conflict of interest.
